# Comparing Prehospital Time Among Pediatric Poisoning Patients in Rural and Urban Settings

**DOI:** 10.5811/westjem.33507

**Published:** 2025-05-23

**Authors:** Aaron T. Phillips, Michael Denning, Em Long-Mills, Dmitry Tumin, Jennifer Parker-Cote, Kathleen Bryant

**Affiliations:** * Brody School of Medicine, Department of Medical Education, Greenville, North Carolina; † Brody School of Medicine, Department of Clinical and Educational Scholarship, Greenville, North Carolina; ‡ Brody School of Medicine, Department of Emergency Medicine, Greenville, North Carolina

## Abstract

**Objectives:**

Barriers to healthcare in rural areas can delay treatment in pediatric patients who have experienced poisoning. We compared emergency medical services (EMS) response times and EMS-reported delays in responding to pediatric poisoning incidents between rural and urban settings using the 2021 National Emergency Medical Services Information System (NEMSIS).

**Methods:**

The NEMESIS defines rural areas as locations with a population of <50,000, not part of metropolitan areas, while all other locations are classified as urban (metropolitan) areas. In this study we included 11,911 patients (12% rural) <18 years of age who were transported by EMS with a first-responder primary impression of poisoning. We compared study variables using rank-sum tests and chi-square tests. Multivariable analysis of outcomes included quantile regression and logistic regression for continuous data and categorical data, respectively.

**Results:**

The median total prehospital time by EMS was 40 minutes (interquartile range 29–57), and the most common type of delay was scene delay (6%). On multivariable quantile regression, patients transported by rural EMS agencies experienced 6.6 minutes (95% confidence interval 5–8, P<0.001) longer prehospital time than those transported by urban agencies. There were no differences between rural and urban EMS agencies in the occurrence of dispatch, response, scene, and transportation delays.

**Conclusion:**

These results elucidate the need for equitable allocation of resources and training to enhance rural EMS responders. The additional nearly seven minutes translates into greater risk for the human body to remain physiologically unstable and not be optimally treated. Therefore, by integrating targeted interventions to rural pediatric populations, better care can be achieved across all geographic regions. Further research must be conducted to ascertain the specific factors, aside from delays, that result in the disparity between rural and urban prehospital response time.

## INTRODUCTION

Pediatric poisoning can occur from a variety of toxic substances. Children in rural settings have a higher incidence of exposure to poisoning events.^1^,^2^ Additionally, youth in rural populations are more likely to attempt suicide, including intentionally using poisoning as a method.^1^ Some chemicals, such as organophosphate and carbamate pesticides, are commonly used by farm workers, introducing the risk of unintentional poisoning events and especially posing a threat among children in rural areas.^3^

Barriers to healthcare in rural areas may negatively influence the time to treatment of pediatric poisoning events before patients reach a facility with the proper resources to manage these emergencies. Barriers may include limited resources within the community, especially when taking into consideration the shortage of emergency medical services (EMS) personnel in rural areas, further exacerbated by the lasting effects of the COVID-19 pandemic and financial inadequacies of rural programs, which limit care.^4^,^5^ As well as increased transportation barriers, including distance to medical centers, additional barriers include inadequate training of medical personnel to treat the disparities present in rural communities; and professional-to-patient language barriers.^6^ Additionally, there is a potential health outcome disparity produced by longer pediatric patients transport to urban pediatric specialty care emergency departments (ED) compared to rural EDs.

Epidemiological studies have compared the incidence of pediatric poisoning events in rural to urban areas in countries other than the US,^8^,^9^ while US studies to date have focused on poisoning prevention education and the use of EDs rather than contacting poison control centers in cases of pediatric poisoning.^10^,^11^ However, no recent study has compared prehospital responses to pediatric poisoning events in rural and urban populations within the US. Due to the scarcity of current literature and the widespread occurrence of pediatric poisonings, there is a need to investigate the impacts and disparities associated with EMS care for this patient population. Our primary aim was to compare the EMS response time (time from the call being received by dispatch until the responding unit’s arrival at the destination hospital or ED) between pediatric poisoning events transported by rural as compared to urban EMS agencies. Secondary aims included comparing the incidence of dispatch delays, response delays, scene delays, and transport delays between rural and urban settings, as well as analyzing demographic disparities between these two settings.

## METHODS

We used the 2021 National Emergency Medical Services Information System (NEMSIS), a multiagency database of EMS activations in the US that included an entire calendar year of data, January 1, 2021–December 31, 2021. All 50 states participate in voluntary submission of datasets to NEMSIS. Due to the use of deidentified data, this analysis was not considered human subjects research by our institutional review board. We identified pediatric patients 0–18 years of age who had a primary impression of poisoning (*International Classification of Diseases*, 10th Rev, ICD-10) code ranges: T43.641A-T54.0X4A, T55.0X1A-T62.94XA, T64.01XA-T65.814A, and T65.831A-T65.94XA). These ICD-10 codes relate to toxic effects caused by various substances: T43.641A-T54.0X4A covers toxic effects of non-medicinal substances such as organic solvents and corrosive acids and alkalis; T55.0X1A-T62.94XA covers toxic effects of soaps, detergents, pesticides, and poisonous ingested foods; T64.01XA-T65.814A covers toxins produced by mold such as aflatoxins in contaminated food as well as other chemicals like nitroglycerin and benzene derivatives; T65.831A-T65.94XA refers to toxic effects of other substances such as nicotine, carbon disulfide, and any unspecified chemical. We excluded patients >18 years of age, patients not transported by the responding EMS unit, patients not transported by land or air EMS, and patients who died prior to arrival at a hospital or ED. We excluded patients not transported by the responding EMS unit due to their transportation occurring via a different EMS agency or individuals, or they were not transported at all. Additionally, we excluded any patients offered treatment by individuals other than EMS professionals, since bystander intervention may have confounded the measurement of prehospital times. Lastly, we excluded cases with missing data on study variables.

**Population Health Research Capsule**

What do we already know about this issue?

*Rural pediatric poisoning cases face delayed EMS care due to limited resources, longer transport distances, and workforce shortages.*

What was the research question?

*Is there a disparity in EMS prehospital care between rural and urban pediatric poisoning cases?*

What was the major finding of the study?

*On multivariable quantile regression, patients transported by rural EMS agencies experienced 6.6 minutes (95% confidence interval 5–8, P<0.001) longer prehospital time than those transported by urban agencies.*

How does this improve population health?

*Elucidating disparities in rural EMS services highlights the need for targeted interventions to improve rural care access and outcomes in pediatric poisoning cases.*

The primary outcome was total EMS response time, or time from the call being received by dispatch until the unit’s arrival at the destination hospital or ED. The secondary outcomes were incidence of any delays during dispatch, response, time on scene, or transport, as reported by the EMS agency. Delays are identified in the NEMSIS registry through self-reporting by EMS agencies. The exposure is defined as the EMS location, whether urban or rural. Rural areas were defined as any location with a <50,000 population that is not part of a metropolitan area, and all other locations were classified as urban (metropolitan) areas. (Secondary exposures by EMS were not observed in this study).^12^,^13^ County lines are often the borders used to define these areas. The NEMSIS database also classifies based on the US Department of Agriculture Economic Research Service definition. Covariates included patient age, sex, race and ethnicity (non-Hispanic White, non-Hispanic Black, Hispanic or Latino, or none of the above),=; initial patient acuity (critical, emergent, or lower acuity); and EMS transportation method (ground or air).

Per the NEMSIS data dictionary, dispatch delay is defined as ”any time delay that occurs from the time of Personal Safety Answering Point (PSAP) call to the time the unit is notified by dispatch.” Response delays encompass “any time delay that occurs from the time the unit is notified by dispatch to the time the unit arrived on scene.” A scene delay is “any time delay that occurs from the time the unit arrived on scene to the time the unit left the scene.” In terms of transport, delay is defined as “any time delay that occurs from the time the unit left the scene to the time the patient arrived at the destination.”

We summarized continuous data using medians with interquartile ranges (IQR), and categorical data using counts and percentages. Study variables were compared between rural and urban settings using rank-sum tests or chi-square tests, as appropriate. Multivariable analysis of study outcomes included quantile regression for continuous data, and logistic regression for categorical data. We reported confidence intervals to characterize the accuracy of the regression coefficients. Each regression coefficient for categorical variables in the model represents an estimated change in the outcome when comparing that category to the reference group. Data analysis was completed in Stata/SE 18.0 (StataCorp, LP, College Station, TX), and *P*<0.05 was considered statistically significant We did not complete a power analysis. STROBE guidelines were followed in the presentation of the data.^14^

## RESULTS

The NEMSIS dataset included 31,179 patients 0–18 years of age who had a “provider primary impression” of poisoning. We excluded 6,902 patients who were not transported by the responding EMS unit (either by land or air), or who were not taken to an ED or hospital; we also excluded 2,157 patients who received medication or procedures prior to the responding EMS unit’s care. After excluding 10,209 patients who had missing data on study variables, we retained 11,911 patients for analysis. The most common responder primary impression was poisoning by unspecified drugs, medicaments, and biological substances (T50.90, 59%), poisoning by other drugs, medicaments, and biological substances (T50.99, 28%), and poisoning by unspecified substances (T65.9, 7%). All other ICD-10 codes for poisoning accounted for <1% of cases each. In the entire sample, the median total EMS response time was 40 minutes (IQR 29–57), and the most common type of delay was scene delay (6%), followed by response delay (5%), transportation delay (3%), and dispatch delay (1%).

The sample included 10,498 (88%) cases reported by an EMS agency in an urban location and 1,413 (12%) cases reported by an EMS agency in a rural location.

Table 1 presents the bivariate analysis of poisoning patient characteristics by urban or rural EMS agency location. The EMS agencies in rural locations were more likely to transport female patients than EMS agencies in urban locations, which were more likely to transport male patients, although they had the same median age. Non-Hispanic Whites comprised the majority of patients transported by both rural and urban agencies. However, there were higher proportions of Non-Hispanic Black and Hispanic patients transported by the urban agencies. The acuity of patients also differed between the agency types, with a higher proportion of critical (red) and emergent (yellow) cases transported by rural agencies, whereas a greater portion of lower acuity (green) was transported by urban agencies.

Moreover, the proportion of patients transported by air was higher in rural areas compared to urban areas. Total time prior to hospital arrival was greater for cases treated by rural EMS, but a greater proportion of cases treated by urban EMS experienced on-scene delays. With large samples and a non-parametric test, it is possible to attain high levels of statistical significance even if the medians happen to be identical, since the non-parametric test used here is not a test of the medians alone but of the entire distribution in each group.

Table 2 presents the results of the multivariate analysis of EMS time prior to arriving at the hospital by patient characteristics. Each variable category has an individually separate reference (ie, male is the reference for sex, and critical red is the reference for acuity). Patients transported by rural EMS agencies experienced a longer time prior to hospital arrival compared to those transported by urban agencies (6.6minutes (5.2–8.0), <0.001). The 6.6-minute difference is a coefficient from a multivariable quantile regression, as seen in the first row of Table 2. As with all multivariable regression coefficients, it is not based on a difference between one pair of values, but represents the estimated difference associated with a unit change in the independent variable, after adjusting for other variables shown in Table 2. Prehospital times were longer among patients who were female, non-Hispanic White, patients with emergent and low-acuity levels, and patients transported by air. Prehospital times were shorter for those who were older, non-Hispanic Black, Hispanic, or critical (red) acuity.

Table 3 presents the multivariate analysis of different EMS delays by patient characteristics. We used logistic regression for dichotomous secondary outcomes (occurrence of each type of delay). Because the statistical method was the same for all secondary outcomes, we combined them to reflect within this table. Rural agencies experienced no difference in odds of experiencing dispatch, response, on-scene, and transport delays compared to urban agencies. Non-Hispanic Black patients showed higher odds of dispatch delay compared to non-Hispanic White patients, while both non-Hispanic Black and Hispanic patients experienced elevated odds of response and on-scene delays. Additionally, individuals categorized as “other” ethnicities faced higher odds of both dispatch and response delays, with patients of lower acuity (green) more likely to experience dispatch delay but less likely to encounter on-scene delay compared to critical (red) cases, and female patients exhibiting higher odds of transportation delay than male patients, along with older patients having increased odds of transportation delay compared to younger patients.

## DISCUSSION

Our study clarifies the disparities in response times and delays of EMS between rural and urban settings for pediatric poisoning events. Pediatric poisoning events are more prevalent in rural communities and have both short- and long-term impacts on pediatric health outcomes.^3^,^15^,^16^ Our study’s findings highlight the challenges rural communities face in accessing timely emergency care for pediatric poisoning patients. Rural communities often face challenges such as longer transportation distances and limited healthcare resources, exacerbating health outcomes. In our study we found that pediatric poisoning patients transported by rural EMS agencies experienced a longer time prior to hospital arrival compared to those transported by urban agencies, with no difference in the incidence of delays.

Pediatric patients transported by rural EMS agencies experienced longer intervals before reaching a hospital than those transported by urban agencies. These delays could lead to worsened patient outcomes, both short and long term.^16^ The findings are consistent with a previous study, which noted rural EMS agencies required longer response times compared to urban agencies, often performed less advanced cardiopulmonary resuscitative efforts, and the patients were presumably alive or having a return of circulation after a cardiac arrest.^17^ Moreover, rural communities face socioeconomic disadvantages due to a focus on agriculture work and decreased population. The decreased socioeconomic status of a county increases delays in EMS response, primarily due to the lack of nearby hospitals caused by financial instability within these communities, thus increasing transportation delays.^18^ In addition, these time disparities may exacerbate the potential health outcome disparity experienced by pediatric poisoning patients’ transport to urban pediatric specialty care EDs compared to rural EDs. The limited in-hospital expertise combined with added prehospital time are two additional barriers to optimal care. As supported by previous studies, our findings are applicable internationally.^19^

Furthermore, in our secondary analysis we investigated demographic variations related to EMS delays. Non-Hispanic Black and Hispanic patients had increased occurrences of on-scene and response delays yet experienced shorter time prior to the hospital arrival. An article by Young articulates shorter EMS response times but longer ED lengths of stay for Hispanic farmers.^20^ Black patients were shown to have shorter response times among EMS, specifically for cardiac arrest yet were more likely to be deceased upon EMS arrival compared to non-Hispanic Whites.^21^ Pediatric poisoning patients with lower acuity levels were disproportionately affected by dispatch delays and increased EMS time-to-hospital arrival compared to critical acuity patients. The EMS dispatchers require increased time to determine the acuity with lower acuity patients or higher acuity patients.^22^

Efforts to mitigate EMS delays should incorporate multifaceted strategies, including recruitment of EMS personnel and addressing financial deficiencies in rural programs. This would allow for better compensation of prehospital professionals such as EMS personnel, thus attracting more people to the position, and for contributions to better training and education for trainees hoping to practice in rural areas. Other methods of improvement of response times include focused training including route optimization in rural areas, improvement of coordination with dispatch centers, cultural competence and community relations, and better prehospital assessment and stabilization training. This improvement will better suit EMS personnel in their efforts to benefit response time and reduce delays. Improving community systems and streamlining dispatch processes can reduce response times and optimize rescue utilization.

The Joint Committee on Rural Emergency Care proposes expanding transportation methods, integrating EMS with community healthcare, and enhancing workforce training.^23^ The National Advisory on Rural Health and Human Services aims to increase timely access to care by reducing response times, improving training and recruitment, and expanding telehealth services.^24^ Recent studies suggest policy changes to extend transport cutoff times for pediatric trauma patients to Level I pediatric trauma centers.^25^ These initiatives promise timely emergency care access, particularly benefiting pediatric patients, and provide opportunities for quality improvement with rural healthcare delivery to enhance outcomes.

Additionally, this article lays the foundation for future research endeavors. As our study broadly includes any substance that can incite a poisoning event, specific toxidromes and their effects on the body are not explored. Further research must be conducted to determine outcomes of delayed treatments in specific poisoning events and means to address these disparities in rural areas so that treatment may become more equitable. Moreover, additional research investigating the nuance between increased delays yet shorter total times for non-Hispanic Black and Hispanic pediatric patients is warranted.

## LIMITATIONS

Limitations of our study include the inability to verify the accuracy of the information included in the NEMSIS by the reporting EMS agencies. The NEMSIS receives information via voluntary submission by EMS agencies. The exclusion of pediatric patients who suffered a poisoning event but were not transported by EMS or who died prior to arrival at a hospital or ED presents a selection bias and may affect the generalizability of the information provided. This may cause an underestimation of the severity and true incidence of pediatric poisoning events and skew the data toward less severe cases. The data also does not capture patient outcomes, such as length of stay or specific resuscitation efforts.

The lack of granular data input into the NEMSIS database results in a small, unreliable sample. Due to this, we were unable to determine whether the initial poisoning event was intentional or unintentional. Although various forms of poisoning events were evaluated, there was no inclusion of specific poisons. Finally, the data did not include any follow-up information after patients were admitted to a hospital, preventing the analysis of hospital outcomes. The practice of EMS is expected to progress and introduce advancements in care that may produce additional confounding variables and alter the interpretation of the information presented in this article.

## CONCLUSION

Our study underscores a disparity in prehospital response times between rural and urban communities for pediatric poisoning events, highlighting an area of inequality to address within rural settings. This study provides insight into further research areas to investigate the clinical significance and potential outcome disparities associated with the added prehospital time. Efforts to mitigate EMS delays should encompass various strategies, including equitable resource allocation and enhanced training programs. Collaborating with stakeholders can help overcome systemic barriers. Advancing our understanding of prehospital care can ensure equitable access to emergency medical services for all pediatric patients. Future research should explore additional factors influencing EMS response times, including socioeconomic factors and healthcare infrastructures, through longitudinal studies. This ongoing research can inform evidence-based practices and policies, with the goal of equitable access to timely emergency services and safeguarding the health of youth in rural and urban communities.

## Figures and Tables

**Table 1:** Bivariate analysis of poisoned patient characteristics by urban or rural EMS agency location (N=11,911).

Variable	Rural n=1,413 patients		Urban n=10,498 patients		P-value
	
	n (%)	Median (IQR)	n (%)	Median (IQR)	
Age (years)	15	(11, 16)	15	(12, 17)	<0.001
Sex					0.004
Male	465 (33%)		3,869 (37%)		
Female	948 (67%)		6,629 (63%)		
Race and ethnicity					<0.001
Non-Hispanic White	996 (70%)		6,111 (58%)		
Non-Hispanic Black	148 (10%)		2,331 (22%)		
Hispanic	82 (6%)		1,427 (14%)		
Other	187 (13%)		629 (6%)		
Acuity					<0.001
Critical – red	128 (9%)		620 (6%)		
Emergent – yellow	703 (50%)		3,886 (37%)		
Lower acuity – green	582 (41%)		5,992 (57%)		
Transported by air	71 (5%)		135 (1%)		<0.001
Time prior to hospital (minutes)		48 (30, 81)		39 (29, 55)	<0.001
Dispatch delay	9 (0.6%)		106 (1%)		0.17
Response delay	68 (5%)		547 (5%)		0.52
On-scene delay	63 (4%)		617 (6%)		0.03
Transportation delay	33 (2%)		283 (3%)		0.42

*IQR*, interquartile range.

**Table 2:** Multivariate analysis of time prior to arriving at the hospital by patient characteristics (n=11,911).

Variable	Total time prior to hospital (Minutes) Coefficient (95% CI)	*P*-value
EMS agency location		
Urban	Ref.	
Rural	6.6 (5.2, 8.0)	<0.001
Age (years)	−0.4 (−0.5, −0.3)	<0.001
Sex		
Male	Ref.	
Female	1.8 (0.9, 2.8)	<0.001
Race and ethnicity		
Non-Hispanic White	Ref.	
Non-Hispanic Black	−3.2 (−4.3, −2.0)	<0.001
Hispanic	−2.6 (−4.0, −1.2)	<0.001
Other	−0.4 (−2.2, 1.4)	0.67
Acuity		
Critical – red	Ref.	
Emergent – yellow	6.2 (4.3, 8.2)	<0.001
Lower acuity – green	5.6 (3.6, 7.5)	<0.001
Transported by air	68.5 (65.0, 72.1)	<0.001

*CI*, confidence interval; *EMS*, emergency medical services; *Ref*, reference.

**Table 3:** Multivariate analysis of different emergency medical services delays by patient characteristics (n=11,911).

Variable	Dispatch delay OR (95% CI)	Response delay OR (95% CI)	On-scene delay OR (95% CI)	Transportation delay OR (95% CI)
EMS agency location				
Urban	Ref.	Ref.	Ref.	Ref.
Rural	0.8 (0.4, 1.7)	0.9 (0.7, 1.2)	0.8 (0.6, 1.0)	0.9 (0.6, 1.3)
Age (years)	0.98 (0.95, 1.01)	1.01 (0.99, 1.02)	0.99 (0.84, 1.16)	0.97 (0.95, 0.99)^**^
Sex				
Male	Ref.	Ref.	Ref.	Ref.
Female	1.0 (0.7, 1.5)	1.1 (0.9, 1.3)	1.0 (0.8, 1.2)	1.6 (1.2, 2.1) ^***^
Race and ethnicity				
Non-Hispanic White	Ref.	Ref.	Ref.	Ref.
Non-Hispanic Black	4.6 (3.0, 7.1) ^***^	1.5 (1.2, 1.8)^***^	1.2 (1.0, 1.5)^*^	1.1 (0.8, 1.4)
Hispanic	1.5 (0.8, 3.0)	1.4 (1.1, 1.8)^**^	1.4 (1.2, 1.8)^***^	1.3 (1.0, 1.8)
Other	2.6 (1.3, 5.3)^**^	1.7 (1.2, 2.2)^***^	1.2 (0.9, 1.7)	0.8 (0.5, 1.3)
Acuity				
Critical – red	Ref.	Ref.	Ref.	Ref.
Emergent – yellow	1.6 (0.5, 5.3)	1.0 (0.7, 1.4)	0.6 (0.5, 0.9)^**^	0.8 (0.5, 1.2)
Lower acuity – green	3.4 (1.0, 11.0) ^*^	1.0 (0.7, 1.4)	0.7 (0.5, 0.9)^**^	0.8 (0.5, 1.3)
Transported by air	1.9 (0.5, 8.4)	1.3 (0.7, 2.3)	1.1 (0.6, 2.0)	0.5 (0.2, 1.6)

Significance levels (^*^, *P*=<0.05; ^**^, *P*=<0.01; ^***^, *P*=<0.001)

*CI*, confidence interval; *OR*, odds ratio; *Ref*, reference.
